# Experience sharing, emotional reciprocity, and turn-taking

**DOI:** 10.3389/fpsyg.2015.00450

**Published:** 2015-04-14

**Authors:** Melisa Stevanovic, Anssi Peräkylä

**Affiliations:** Finnish Centre of Excellence in Intersubjectivity in Interaction, Department of Social Research, University of Helsinki, Helsinki, Finland

**Keywords:** turn-taking, emotion, experience sharing, emotional contagion, conversation analysis

## Abstract

In this perspective article, we consider the relationship between experience sharing and turn-taking. There is much evidence suggesting that human social interaction is permeated by two temporal organizations: (1) the sequential framework of turn-taking and (2) the concurrent framework of emotional reciprocity. From this perspective, we introduce two alternative hypotheses about how the relationship between experience sharing and turn-taking could be viewed. According to the first hypothesis, the home environment of experience sharing is in the concurrent framework of emotional reciprocity, while the motivation to share experiences is in tension with the sequential framework of turn-taking. According to the second hypothesis, people’s inclination to coordinate their actions in terms of turn-taking is motivated precisely by their propensity to share experiences. We consider theoretical and empirical ideas in favor of both of these hypotheses and discuss their implications for future research.

## Sequentiality and Concurrency in Human Social Interaction

In recent years, there has been an increasing number of conversation analytic studies explicating the social organization of, and the highly ordered interactional tasks performed by, emotional expressions in social interaction (e.g., Peräkylä and Sorjonen, 2012). This perspective paper seeks to further this line of research by considering how expressions in the service of what we call experience sharing are embedded in the most primordial temporal organizations of interaction.

Many naturally occurring interactions call for individuals to coordinate their actions in terms of turn-taking. This happens especially in the context of language use: the principle of one participant talking at a time (Sacks et al., 1974) allows humans to communicate complex thoughts and intentions. In conversation, social actions (e.g., proposals, offers, and invitations) and their responses (e.g., acceptances and rejections) are organized in terms of successive turns at talk. As pointed out by Schegloff (1988, pp. 98–99), turn-taking enables humans to pursue stable trajectories of action and responsive action. This arrangement will be referred to as the *sequential framework of turn-taking*. It denotes not only the temporal but also the conditional relationship between participants’ interactional moves.

In addition to the sequential framework of turn-taking, human interactive conduct is permeated by another temporo-conditional arrangement—something that we call the *concurrent framework of emotional reciprocity*. The literature on “emotional contagion” suggests that humans have an automatic tendency to mimick other people’s non-verbal emotional expressions, which affects the emotional experience of the mimicking person (Hatfield et al., 1993, 1994; Dimberg, 2007). This happens as a result of afferent feedback generated by elementary motor mimicry, which produces a simultaneous emotional match independently of people’s cognitive abilities to understand what is going on in the mind of the other (Carr et al., 2003; Leslie et al., 2004; Barresi and Moore, 2008; Decety and Meyer, 2008). From this perspective, two participants’ interactional moves are, by definition, connected by a causal relationship (one participant produces an expression first and the other acts in response to him/her), but the actions/expressions take place in a shared time. Overlap of expressions is regular. Regarding the opportunity for expression, the participants are positioned symmetrically, which is reflected in the frequent concurrency of the participants’ interactional moves.

There are different motives that lead humans to interact with each other. In addition to their instrumental communicative goals, humans are also motivated to share experiences about events and things in the environment with their significant others and to become “swept along” by them (Feinman, 1982; Striano and Rochat, 1999; Tomasello, 1999; Hobson and Hobson, 2008; Rochat et al., 2009). This motivation has even been regarded as the force that has driven the evolution of language (Locke, 1996, 2002; Dunbar, 1997). But how does experience sharing relate to the sequential framework of turn-taking, where language use regularly takes place? Could it be that experience sharing is “at home” in the concurrent framework of emotional reciprocity, while being in tension with turn-taking? Or, do humans cast their experience sharing into the system of turn-taking precisely because it supports experience sharing? In what is to come, we will discuss these two alternative hypotheses one after another, with the aim of paving the way for future empirical research on the topic.

## Hypothesis I: Experience Sharing is in Tension with the Sequential Framework of Turn-Taking

Our first hypothesis suggests that the home environment of experience sharing is in the concurrent framework of emotional reciprocity, while there is a tension between experience sharing and the sequential framework of turn-taking.

The first pieces of support for this hypothesis come from the developmental psychological research literature, which suggests there to be automatic resonance processes that allow humans, right from the outset, to bridge their own and others’ experiences. With reference to neonatal imitation, Meltzoff and Brooks (2001) have argued that, in reproducing the behavior of others, infants automatically perceive others as “like me” and thus begin to develop a sense of social connectedness, mutual recognition, and shared experience. In other words, experience sharing has been suggested to emerge “in the guise of emotional contagion” (Brinck, 2008).

Another type of support for our first hypothesis comes from adult interaction, and is provided by the temporal organization of the instances of language use associated with experience sharing. While language use may be anchored in the organization of turn-taking (see e.g., Schegloff, 1996, 2006), still, there is much work suggesting that the moments of experience sharing may be exceptional in this respect (Coates, 1994; Lerner, 2002; Pillet-Shore, 2012; Vatanen, 2014). In conversation analysis, a classic example is provided by Goodwin and Goodwin (1987), who described the sharing of affective stances in the form of concurrent agreeing assessments. Their example involves two conversationalists praising something a mutual friend has baked. One of them says: “Jeff made an asparagus pie, it was so good.” In overlap with the first speaker’s “so,” the co-interactant launches an assessment: “I love it.” Hence, it appears that, in the moments of experience sharing, the concurrent framework of emotional reciprocity colonizes the organization of spoken interaction, leading to the momentary relaxation of turn-taking rules. Also studies within the domain of mother-infant interaction have shown that positive affective expressions tend to coincide with simultaneous vocalizations (Stern et al., 1975; Beebe et al., 1979).

Besides overlapping talk, there are also other ways in which experience sharing, as it were, “surpasses” turn-taking. Face is central here. Speakers may use their facial displays to mark a transition from affectively neutral talk to emotional experience sharing in the middle of their ongoing turns at talk (cf. Iwasaki, 2011). Likewise, a recipient may display an emotional stance toward an actional or a topical element in a speaker’s ongoing turn at talk, thereby inviting the speaker to redirect her utterance production (Kaukomaa et al., in press). Detailed considerations of parallel uses of words and facial displays in moments of experience sharing are thus particularly intriguing.

From this perspective, let us consider Example 1 (taken from Peräkylä and Ruusuvuori, 2006), where one participant’s telling (about a dress code that a mutual friend working in a newspaper needed to conform to) is followed by a shared amusement of both of the participants. Here, the onset and the completion of the smiles take place with one party doing the first move and the other party following. However, between these sequentially organized boundary moves, the participants maintain simultaneous smiles, embodying the sharing of experiences over a lengthy period of time.

**Example 1:**
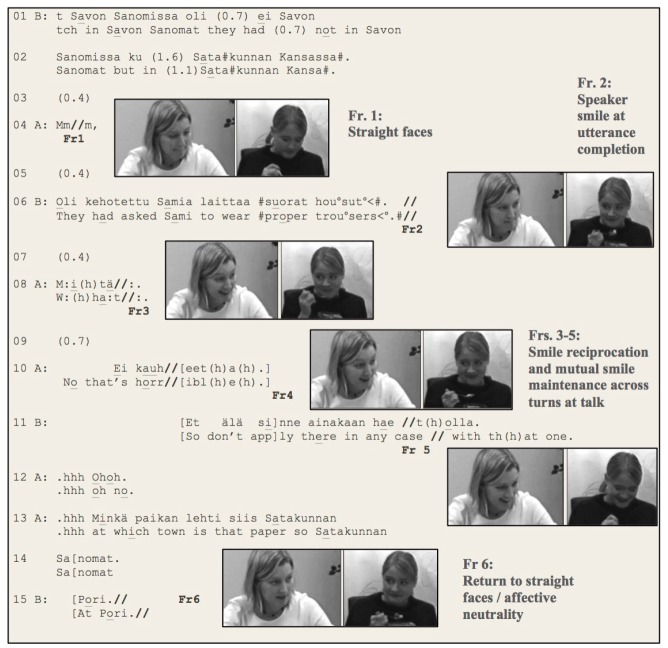


During the most part of the first speaker’s (B’s) telling (lines 1–6) both participants are looking down with straight faces (Frame 1). At the end of the telling, however, they establish mutual gaze and the teller (B) adopts a smiling face (Frame 2). After a gap (line 7), the recipient (A) reciprocates the smile and raises her brows (Frame 3), simultaneously producing an animated verbal response of “ritualized disbelief” (Heritage, 1984, p. 339; line 8). While the interaction has thus far abided to the sequential framework of turn-taking, now, as soon as the mutual smile has been established, the participants’ facial conduct gets detached from the sequential organization of turn-taking as the concurrent framework of emotional reciprocity breaks through. During a moment of “heightened emotive involvement” (Selting, 1994), they maintain their smiles and mutual gaze over a number of turns conveying ostensibly different actions (assessment, line 10; joking advice, line 11; Frame 4). The temporality of the mutual smile goes beyond the turn-taking organization. After this, the participants, however, break their mutual gaze (Frame 5), which is followed by their smiles becoming less intensive. One participant (A) adopts a straight face at the onset of her question (lines 13–14), while the other (B) does the same during her answer to the question (line 15; Frame 6). Thereby, they indicate that the heightened moment of experience sharing is over. While the primary modality of expression surpassing the turn-taking rules in Example 1 was facial expression, and the organization of talk followed turn-taking rules, the overlapping talk (lines 10–11) during the experience sharing should also be acknowledged.

The idea of experience sharing being in tension with the sequential temporality of turn-taking reverberates with certain recent suggestions presented in the conversation analytic literature: Heritage (2011) has described the epistemic dilemmas associated with the “empathic moments in interaction” and Enfield (2011) has suggested that it is turn-taking with its inherent asymmetries that helps to account for the existence of such dilemmas. But even if experience sharing would be in tension with turn-taking, this seems not to be the case for instrumental communication. In her study on university subcommittee meetings, Edelsky (1981) observed that those participants who otherwise made frequent use of overlapping talk still abided to the canonical turn-taking system when their talk was oriented toward the official business of the meeting. The same phenomenon seems to apply also for those emotional expressions that are used to carry out different kinds of instrumental communicative tasks. For example, Heath (1989) showed that, when patients in medical consultations tried to legitimize medical attention to their ailment through cries of pain, these expressions of suffering abided to the organization of turn-taking.

The points detailed above suggest that the home environment for experience sharing might be in the concurrent framework of emotional reciprocity, while experience sharing (unlike instrumental communicative goals) may call for the participants to depart from the sequential framework of turn-taking.

## Hypothesis II: The Sequential Framework of Turn-Taking Serves Experience Sharing

While our first hypothesis questions the relevance of turn-taking for experience sharing, our second hypothesis represents just the opposite view: it suggests that the sequential framework of turn-taking is *not* in tension with experience sharing but, instead, serves it.

Previously, we highlighted the significance of the automatic resonance processes for experience sharing. However, it has been pointed out that the mere reproduction of other people’s behaviors represents a closed loop system: it reflects what is already out there (Rochat and Passos-Ferreira, 2008). So, for people really to relate and share each other’s experiences more fully, these automatic resonance processes need to be embedded in an open system of *contingent* emotional reciprocity. It can thus be argued that contingent emotional reciprocity, and thus also experience sharing, can be facilitated by the sequential framework of turn-taking.

In human ontogeny, the first instantiations of contingent emotional reciprocity appear in the context of alternation between approach and withdrawal tendencies (Beebe and Stern, 1977; Hietanen et al., 2008). Infants have been shown occasionally visually to disengage from their interactive partners and then return to the engagement. Often, this happens in a highly coordinated fashion—that is, when one partner moves from a less engaged phase to a more engaged phase, or vice versa, the other partner responds with a corresponding change in the same direction. Still, these changes are likely to take place within certain time-lags (Cohn and Tronick, 1987). This gives the interaction a sense of one person making a bid of engagement and another person responding to that bid in a positive way.

In the subsequent development of the human infant, the experiences of contingent emotional reciprocity get more nuanced. Already from 2 months on, infants and their caretakers start to look and listen to each other more carefully; producing vocal, facial and gestural responses elicited by the expressed feelings and interests of their interaction partners (Spitz and Wolf, 1946; Trevarthen, 1979; Stern, 1985; Cohn and Elmore, 1988; Rochat and Passos-Ferreira, 2008). During these monitoring processes, the infants gradually develop expectations for how the interaction is likely to proceed; for example, they learn to expect that, following an emotional bid on their part, be it via a smile, gaze, or frown, the other will respond in return (Sagi and Hoffman, 1976; Meltzoff and Moore, 1977; Wolff, 1987; Sroufe, 1996; Rochat, 2001). Compared to the automatic resonance processes, the reliance on social expectations is risky but, when successful, likely to result in a powerful experience of shared emotion (Rochat et al., 2009).

From this perspective, let us consider Example 2, where an 11-month-old girl, Nea, prompts a prominent instance of experience sharing. First, she looks at her parents, assuring their attention. Thereafter, she puts a funny grimace on her face, thus prompting her parents’ to laugh heartfeltly. Finally, she joins in the laughter, expressing a high level of positive arousal. The sharing of experience is organized in successive turns. This turn-taking organization of sharing builds on the child’s capacity to anticipate her significant others’ reactions to her behavior, while there is a genuine possibility that the parents will not behave as expected.

**Example 2:**
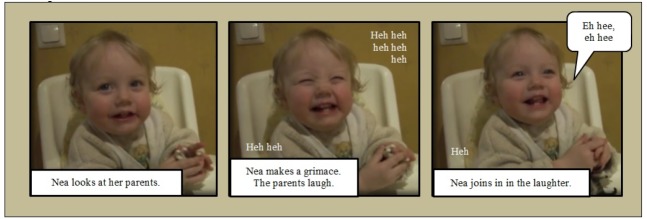


Example 2 demonstrates how a turn-taking structure, where expressions are organized as distinct temporal units that follow each other, provides for experiences of contingent emotional reciprocity. By making a grimace, Nea, took on trust that her parents would recognize what she was up to; in their “next turn” (laughter), the parents displayed that they indeed did; and, the correctness of the parents’ interpretation was confimed by Nea, through her final laughter. Here, turn-taking enables the participants to establish meaningful linkages between their behaviors. While each behavior occupies a forward-looking status, raising expectations for what is to happen next (on conditional relevance, see Schegloff, 2007, p. 20), each response to such behaviors occupies a backward-looking status as something that was invoked by what just occurred (on the next-turn proof procedure, see Sacks et al., 1974, pp. 728–729). It is thus the dynamic interplay between expectations and their overflowing satisfaction that provides a scaffolding for a possible escalation of emotion displays (each participant endorsing the affective aspect of their co-participants’ turns), which may generate particularly intensive instances of experience sharing. Also the conversation analytic research on emotional expression provides support for our second hypothesis. This line of research has shown that the participants may refrain from the immediate reciprocation of contagious emotional expressions, such as laughter and crying (Hepburn and Potter, 2012; Shaw et al., 2013). Instead, these expressions seem to be regulated by the turn-by-turn sequential organization of interaction. For example, the recipients of complaint stories (Couper-Kuhlen, 2012) and news deliveries (Maynard and Freese, 2012) have been shown to produce their emotional responses at the completion of the news delivery or narrative, rather than immediately after the tellers’ emotional displays. Likewise, surprise tokens such as *wow*, *gosh*, *oh my good*, *ooh*, *phew* have been described as interactionally organized performances, interactional achievements, instead of automatic emotional eruptions (Wilkinson and Kitzinger, 2006; see also Heath et al., 2012). It could be precisely the sensitivity of emotional expression to the sequential framework of turn-taking that enables the participants to display their willingness to grasp—as fully as possible—the particular experience that their co-participants are about to share.

Finally, we may turn the table around and ask whether experience sharing can also serve turn-taking. This possibility appears relevant with reference to the Goffmanian idea of the ubiquitous insecurity of people in social interaction—a theme that runs all through his work (see e.g., Goffman, 1955; Rawls, 1987). In social interaction, by every turn that a person takes, s/he claims being worthy of other’s attention and calls for others to recognize this claim (Goffman, 1955, pp. 9–10), while there is always a possibility that this claim will *not* be recognized by others (Stevanovic, 2015; Peräkylä, in press). The emotionally secure framework of experience sharing with significant others, however, provides an embarrassment-free site for the practicing of making such claims and for the acquisition of the more asymmetric responsibilities that the sequential framework of turn-taking enforces the participants to assume.

In sum, the coordination of actions in terms of turn-taking enables forms of experience sharing that the concurrent organization could not afford. This suggests that one motivation underlying the human propensity to coordinate their actions in terms of turn-taking could be the human propensity to share experiences. Even more, there might be a bidirectional linkage of enhancement between these two propensities.

## Consequences for the Study of Turn-Taking

We have now considered two alternative hypotheses about how the relationship between experience sharing and turn-taking could be viewed. What consequences would these imply for the study of turn-taking?

According to our first hypothesis, the home environment of experience sharing is in the concurrent framework of emotional reciprocity, while there is a tension between experience sharing and the sequential framework of turn-taking. If this hypothesis is valid, then there must be evidence that, not only does a lesser amount of experience sharing lead to a greater amount of turn-taking, and vice versa, but also, that turn-taking obstructs experience sharing. Furthermore, if turn-taking is there to facilitate instrumental communication (instead of experience sharing), then we would expect that there would be a tension between overlapping vocalizations and effective instrumental communication. While there is some evidence for the tension between turn-taking and experience sharing (Enfield, 2011; Heritage, 2011; Vatanen, 2014), the potentially problematic combination of overlapping vocalizations and effective instrumental communication has rather been taken for granted than really unpacked through empirical investigation (cf. Stevanovic and Frick, 2014). One further challenge from the perspective of this hypothesis would be to account for the occurrences of overlapping talk serving instrumental purposes.

According to the second hypothesis, turn-taking is in the service of experience sharing. In allowing the participants to engage in increasingly complex forms of joint action, and, hence, in ever more exciting shared experiences, there seems to be no tension between turn-taking and experience sharing. From this perspective, there seems to be a developmental continuity between the early infant-caretaker interactions governed by emotional reciprocity and the later, more complex forms of social interaction. If this hypothesis is valid, then there must be evidence that the instances of experience sharing cast in the sequential framework of turn-taking can, in principle, reach at least the same level of intensity as those occurring in the context of overlapping talk. Moreover, one would need to show that occurrences of experience sharing and instrumental goal-pursuit would be relatively evenly distributed between the instances of overlapping talk and talk abiding to the norms of turn-taking.

No matter which of the above hypothese is more valid than the other, the relationship between experience sharing and turn-taking is something worth further investigation—something that this paper has sought to highlight.

### Conflict of Interest Statement

The authors declare that the research was conducted in the absence of any commercial or financial relationships that could be construed as a potential conflict of interest.
